# Novel risk genes for systemic lupus erythematosus predicted by random forest classification

**DOI:** 10.1038/s41598-017-06516-1

**Published:** 2017-07-24

**Authors:** Jonas Carlsson Almlöf, Andrei Alexsson, Juliana Imgenberg-Kreuz, Lina Sylwan, Christofer Bäcklin, Dag Leonard, Gunnel Nordmark, Karolina Tandre, Maija-Leena Eloranta, Leonid Padyukov, Christine Bengtsson, Andreas Jönsen, Solbritt Rantapää Dahlqvist, Christopher Sjöwall, Anders A. Bengtsson, Iva Gunnarsson, Elisabet Svenungsson, Lars Rönnblom, Johanna K. Sandling, Ann-Christine Syvänen

**Affiliations:** 1 Department of Medical Sciences, Molecular Medicine and Science for Life Laboratory, Uppsala University, Uppsala, Sweden; 2 Department of Medical Sciences, Rheumatology and Science for Life Laboratory, Uppsala University, Uppsala, Sweden; 3 Rheumatology Unit, Department of Medicine, Karolinska Institutet, Karolinska university hospital, Stockholm, Sweden; 4 Department of Public Health and Clinical Medicine/Rheumatology, Umeå University, Umeå, Sweden; 5 Lund University, Skåne University Hospital, Department of Clinical Sciences, Rheumatology, Lund, Sweden; 6 AIR/Rheumatology, Department of Clinical and Experimental Medicine, Linköping University, Linköping, Sweden; 7 Science for Life Laboratory (SciLifeLab), Department of Biosciences and Nutrition, Karolinska Institutet, Solna, Sweden

## Abstract

Genome-wide association studies have identified risk loci for SLE, but a large proportion of the genetic contribution to SLE still remains unexplained. To detect novel risk genes, and to predict an individual’s SLE risk we designed a random forest classifier using SNP genotype data generated on the “Immunochip” from 1,160 patients with SLE and 2,711 controls. Using gene importance scores defined by the random forest classifier, we identified 15 potential novel risk genes for SLE. Of them 12 are associated with other autoimmune diseases than SLE, whereas three genes (*ZNF804A*, *CDK1*, and *MANF)* have not previously been associated with autoimmunity. Random forest classification also allowed prediction of patients at risk for lupus nephritis with an area under the curve of 0.94. By allele-specific gene expression analysis we detected *cis*-regulatory SNPs that affect the expression levels of six of the top 40 genes designed by the random forest analysis, indicating a regulatory role for the identified risk variants. The 40 top genes from the prediction were overrepresented for differential expression in B and T cells according to RNA-sequencing of samples from five healthy donors, with more frequent over-expression in B cells compared to T cells.

## Introduction

Systemic lupus erythematosus (SLE) is a chronic autoimmune disease with complex etiology. SLE is considered as a model for systemic autoimmune diseases, in which most organ systems of the human body can be affected. SLE is characterized by the presence of autoantibodies, immune complex formation, and organ inflammation. The disease phenotype varies between individual patients from relatively mild manifestations of skin and joints to organ-threatening renal involvement (lupus nephritis)^1^.

Genome-wide association studies (GWAS) have identified over 60 genetic loci that confer risk for SLE^2^, but a large proportion of the genetic contribution to SLE susceptibility still remains unknown. Although the genetic background of specific manifestations of SLE is less well known than that of SLE in general, several single nucleotide polymorphisms (SNPs) have also been associated with the subgroup of patients with lupus nephritis^3, 4^.

SNPs that reach genome-wide, or close to genome-wide, significance in genetic association studies have been compiled in the GWAS catalog^5^. Using the collective information from many single nucleotide polymorphisms (SNPs) it should be possible to identify novel disease-associated genes. There are multiple machine learning methods that could be applied to SNP genotype data, such as logistic regression, artificial neural networks^6^, support vector machines^7^, and random forests^8^. Information from the GWAS catalogue has been used for predicting risk of 18 common diseases using a logistic regression model evaluated on SNP allele frequency and odds ratios^9^, but SLE was not included in this study.

In the current study our aim was to use machine learning based on random forests to design a SNP genotype classifier to predict risk of SLE and to predict previously unknown genes and genetic variants that confer risk of SLE. Out of the multiple prediction algorithms available, we chose to use the random forests machine learning method^8^, as this method has been shown empirically to perform well in many scenarios where predictions are needed^10^ and is applicable to genetic association data^11, 12^. We used genotype data from the Immunochip^13, 14^ (Illumina), which targets about 200,000 SNPs in genes that are relevant for diseases of the immune system, to predict disease status in a large set of Swedish patients with SLE and controls and to identify risk genes for SLE. A high disease probability from the classifier indicates higher genetic risk for SLE for an individual compared to the general population, and a high gene importance score in the model indicates a gene region that contains SNP alleles that confer risk of SLE.

SLE is a systemic autoimmune disease characterized by activated T cells and autoantibody production by B cells. We therefore applied analysis of allele-specific gene expression (ASE)^15^ and RNA sequencing to assess functions of the candidate genes for SLE predicted by the random forest algorithm, in B and T cells from peripheral blood of healthy donors. The genotype data from the Immunochip combined with cell type-specific gene expression and ASE in B and T cells yield information on the regulation of gene expression of the putative risk genes for SLE defined by the random forest algorithm.

## Results

### Prediction of genetic risk for SLE by random forests

**Figure 1:**
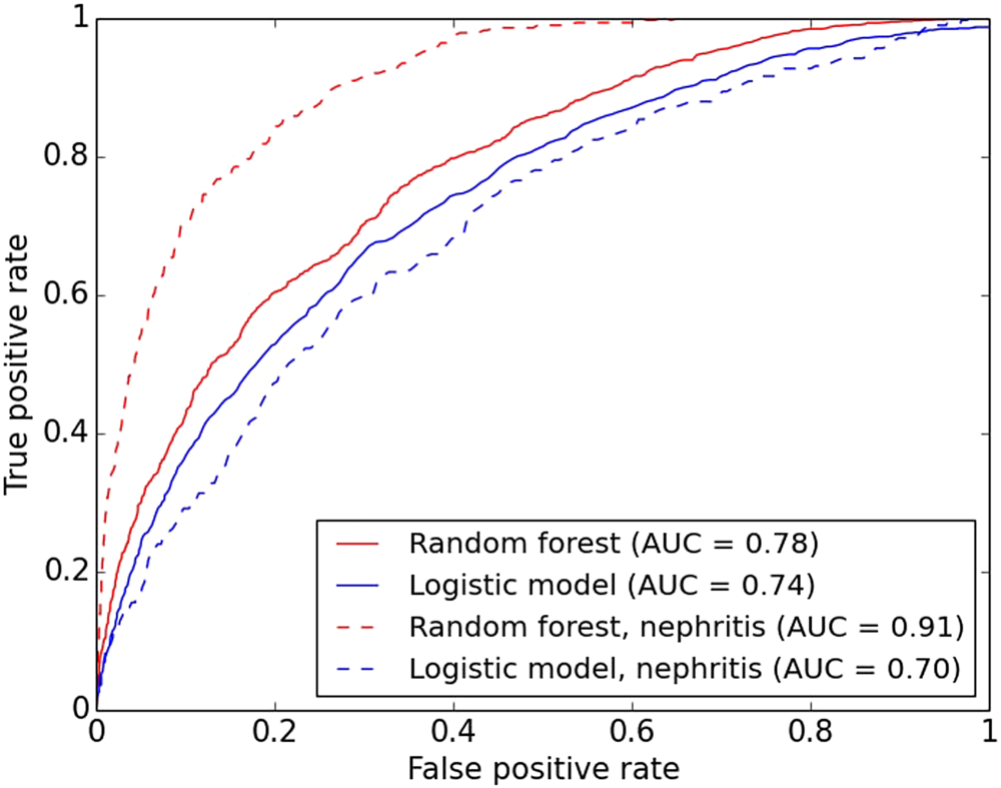
Prediction accuracy. Prediction accuracy measured by the area under the curve (AUC) using genotype data from the Immunochip. All data from 1,160 SLE patients and 2,711 controls were used for the prediction of SLE disease status by random forests (RF) and using a risk score based on the single SNP association analysis. The random forest classification was also applied to the subgroup of the SLE patients diagnosed with lupus nephritis (n = 274) together with all control samples.

We used machine learning based on random forests to design a SNP genotype classifier to discern between patients with SLE and healthy individuals. For this purpose we used quality controlled genotype data for 134,523 SNPs from the Immunochip (Illumina) located in or close to 12,500 genes related to the immune system from 1,160 patients with SLE and 2,711 healthy controls. The random forest classifier yields a probability that a sample originates from a patient with SLE for each individual. This probability value was used to calculate the area under curve (AUC) as a measure of the prediction accuracy. The AUC, based on a receiver operating characteristic (ROC) curve^16^, offers the advantage of combining the specificity and sensitivity measures into one accuracy without setting a fixed threshold for evaluation of the accuracy. The AUC can range from 0 to 1, where an AUC-value of 0.5 equals a random prediction and an AUC of 1 represents a perfect prediction. The AUC for the random forest prediction of SLE was 0.78 (Fig. 1), which in comparison with the AUC of 0.74 for the logistic model is a significant improvement (p-value 0.0028, DeLong’s test which calculates the significance of the difference between two dependent ROC curves based on the same sample set)^17^.

The explained heritability for different models was calculated using the AUC value for each model in conjunction with the disease prevalence and the sibling recurrence risk for SLE. The SNPs reported in the GWAS catalog account for 5% of the heritability for SLE, compared to 11% using the genetic risk score obtained by logistic regression of the case-control association data from our study, and 16% obtained using the random forest model for our data.

The SLE patients with lupus nephritis is a clinically more homogeneous subgroup with a severe manifestation of SLE. As lupus nephritis is the only manifestation of SLE defined by the 1982 American College of Rheumatology (ACR) criteria^18^ for which there are associated SNPs that reach genome wide significance in the GWAS catalog, we constructed a random forest classifier also for this subgroup of patients. Analysis of the 274 samples from SLE patients with lupus nephritis in our cohort yielded a high success in the random forest prediction, with an AUC of 0.91 compared to 0.78 for prediction of SLE in the whole cohort. Predictions using the risk score calculated from the regular SNP association analysis (logistic regression) for the same patients with lupus nephritis yielded a significantly lower AUC of 0.70 (Fig. 1, p-value < 2.2E-16, DeLong’s test). The estimated explained heritability for lupus nephritis is 47% according to the random forest model.

### Prediction of risk genes for SLE by random forests

We used the random forest algorithm to calculate “importance scores” based on the genotype data from the Immunochip. This score describes to what extent a gene region confers risk of SLE based on the classification performance of the SNPs in the gene region (for more details, see Materials and Methods). The gene importance scores for all genes from the random forest prediction are listed in Supplementary Table S1. As can be seen in Supplementary Fig. S1, the gene importance scores follow a log-linear distribution with a low slope of Y = 10^0.0003*X^ corresponding to a 0.07% increase in score for each rank from the lowest rank to approximately gene rank 500, after which the slope is 3.3 times steeper (Y = 10^0.001*X^), and at around gene rank 40 the slope is additionally 8.1 times steeper (Y = 10^0.008*X^). Based on this observation, together with the fact that there are as many as 25 known SLE associated genes among the top 40 predicted risk genes, we chose the 40 top genes from the random forest prediction for further investigation.

Of the 40 genes with the highest gene importance scores, 12 genes, *PSMG1*, *PTGER4*, *CPEB4, EGR2, RFX3, IL1R1*, *LRRK2*, *GPR183*, *ZMIZ1*, *ELMO1*, *TNFSF11*, *SATB2* are associated with other autoimmune diseases than SLE according to the GWAS catalog (Table 1). The random forest classification predicted *ZNF804A*, *ANK3* and *DOCK3* that have so far not been connected to SLE or any other autoimmune disease to be risk genes for SLE (Table 1). However, in the regions nearby the *ANK3* and *DOCK3* gene there are genes implicated in SLE based on functional evidence rather than associations listed in the GWAS catalog^19, 20^. Based on their function, as discussed below, the most likely candidate gene for SLE risk in the *ANK3* gene region is *CDK1* and for the *DOCK3* gene region *MANF* is a likely candidate gene. Notably, the *ZNF804A* and the *ANK3*/*CDK1* genes obtained the fifth and sixth highest gene importance scores in the random forest classification, and thus the probability for them being true risk genes for SLE can be considered as high, since all but two other genes reaching rank 20 or higher were known SLE genes identified by GWAS.

**Table 1:** Top 40 risk genes for SLE identified by random forest prediction using Immunochip genotype data from SLE patients and controls.

Predicted genes^1^	Gene importance score^4^	Association with autoimmune diseases in the GWAS catalog^5^	Differential expression in B and T cells^8,9^
*BLK*	51.8	SLE, RA, KD, pSS	B > T***
*CLEC16A*	49.2	SLE, IBD, UC, T1D, MS, CD, Psoriasis	B > T
*STAT4*	39.8	SLE, UC, IBD, RA, CD, pSS, Celiac, PBC	T > B***
*ETS1*	33.5	SLE, RA, Celiac, Psoriasis	T > B
*ZNF804A*	33.4	New	B > T***
*ANK3* ^3^ *CDK1* ^2^	33.0	New	T > B
*BANK1*	30.5	SLE, IBD, CD	B > T***
*PSMG1*	27.5	IBD, UC, CD, AS	T > B
*TNIP1* ^3^	27.2	SLE, IBD, Psoriasis	B > T
*PLEKHH2 THADA* ^2^	26.7	SLE^6^, CD, IBD, MS	Low
*TPI1P2 TNPO3* ^2^ *IRF5* ^2^	26.6	SLE, PBC, pSS	Low
*IKZF1* ^3^	25.9	SLE, IBD, CD, UC	T > B
*PTGER4*	24.4	IBD, CD, UC, AS, MS	T > B
*CD44*	23.9	SLE, Vitiligo	T > B
*IRF5*	23.1	SLE, UC, IBD, RA	B > T***
*IL2RA*	22.8	SLE^6,7^, IBD, CD, T1D, RA, MS, Vitiligo	T > B
*TNFSF4*	21.6	SLE, CD, RA, Celiac, MS	Low
*SLC15A4*	20.4	SLE	B > T
*IL12A-AS1 IL12A* ^2,3^	20.1	SLE^6^, Celiac, PBD, MS, pSS	Low
*HIP1*	19.7	SLE	B > T
*XKR6*	18.2	SLE	Low
*CPEB4*	17.8	IBD, CD	B > T*
*ZNF365 EGR2* ^2^	17.6	IBD, CD, UC, RA	T > B*
*THADA*	17.2	SLE^6^, IBD, CD, MS	B > T
*GLIS3 RFX3* ^2^	16.7	T1D	Low
*NCF2* ^3^	16.7	SLE	B > T
*PHRF1* ^3^	16.6	SLE	T > B
*PAPOLG*	16.4	SLE^6^, CD, RA, Psoriasis	T > B
*IL1R1*	16.4	IBD, UC, CD, AS	Low
*LRRK2*	16.1	IBD, CD, UC	B > T***
*UBAC2 GPR183* ^2^	15.8	IBD, CD	T > B
*ZFP36L2 THADA* ^2^	15.4	SLE^6^, IBD, CD, MS	T > B
*PVT1*	15.4	SLE^6^, RA, MS	T > B
*ZMIZ1*	15.3	IBD, CD, MS, Vitiligo, Psoriasis	Low
*ELMO1*	14.9	CD, RA, PBC, Psoriasis	B > T
*WDFY4*	14.9	SLE, RA	B > T***
*AKAP11 TNFSF11* ^2^	14.8	IBD, CD	B > T
*DOCK3 MANF* ^2^	14.7	New	Low
*SATB2*	14.7	UC, IBD	Low
*IRF8*	14.6	SLE, IBD, UC, RA, PBC, CD	B > T***

^1^Human leukocyte antigen (HLA) genes not included, ^2^Alternative candidate autoimmunity gene in the region reported in the GWAS catalog or functional studies, ^3^Cis-regulatory SNPs with significant association with allele-specific gene expression in B or T cells, ^4^The random forest generates SNP importance scores based on the importance of each SNP for the prediction. The SNP scores are summed up over a gene region to obtain the final gene importance score, ^5^SLE = systemic lupus erythematosus, RA = rheumatoid arthritis, IBD = inflammatory bowel disease, CD = Crohn’s disease, T1D = diabetes mellitus type 1, MS = multiple sclerosis, PBC = primary biliary cirrhosis, UC = ulcerative colitis, KD = Kawasaki disease, Celiac = Celiac disease, AS = Ankylosing spondylitis, pSS = primary Sjögren’s syndrome, New = previously unknown SLE risk gene, ^6^Langefeld, C. D. *et al*. Transancestral mapping and genetic load in systemic lupus erythematosus, submitted manuscript, ^7^Evidence of SLE association from literature^44^, ^8^Genes are annotated according to their expression level in B or T cells based on RNA-sequencing data, ^9^Low = Expression below 1 fragments per kilobase of exon per million fragments mapped (FPKM) for both cell types, ^*^Bonferroni corrected p-value < 0.05, ^***^Bonferroni corrected p-value < 0.001.

The putative novel risk gene for SLE, *ZNF804A* upregulates the expression of COMT and a coding variant in *COMT* has previously been associated with a slightly increased risk of SLE^21^. Moreover, *ZNF804A* downregulates the expression of PDE4B^22^, a protein involved in inflammatory pathways. In fact, the PDE4B-specific small drug inhibitor NCS 613 has been shown to have anti-inflammatory properties in PBMCs from both healthy donors and SLE patients and is considered as a complementary strategy for the management of SLE^23, 24^.

*CDK1* is located 30 kb upstream of the longest transcript of *ANK3*. *CDK1* enhances type I IFN signaling by promotion of the type I IFN-induced phosphorylation of STAT1 and up-regulation of the expression of interferon-stimulated genes^19^, which is a hallmark of SLE^25^. Also The SLE associated CDK inhibitors CDKN1A and CDKN1B have been shown to interact with CDK1^26^. Expression of CDK1 is elevated in peripheral blood mononuclear cells (PBMCs) and kidney biopsy specimens from SLE patients and is correlated with the expression of three representative IFN-inducible genes (*IFI27*, *IFIT3*, and *CXCL10*). Additionally, a CDK1 inhibitor was shown to reduce the expression of interferon-stimulated genes in PBMCs from SLE patients and in renal cells from mice with SLE^19^.

*MANF* is located 1 kb downstream of *DOCK3*. Dysfunctional response to unfolded proteins in the endoplasmic reticulum (ER) was found in SLE patients with upregulated levels of MANF. Stress of the ER is closely correlated with inflammation and/or immune diseases. However, it is still unknown whether aberrant ER stress is involved in SLE pathogenesis^20^.

Based on the known involvement of ZNF804A, CDK1 and MANF in important pathways that are affected in SLE, these three genes are strong novel candidate risk genes for SLE. The 12 putative novel SLE genes with associations reported to other autoimmune diseases than SLE in the GWAS catalog are also of great interest due to their potential involvement in the pathogenesis of SLE (Table 1, Supplementary Table S2). Of the 40 genes predicted to confer risk of SLE in the random forest classification, eight are significantly overexpressed in B cells compared to T cells from healthy individuals, while two genes are significantly overexpressed in T cells (Bonferroni corrected p-value < 0.05) (Table 1).

Creating a classifier for patients with lupus nephritis and treating the other SLE patients as a control group, allows identification of genes that distinguish patients with lupus nephritis from other SLE patients. Somewhat surprisingly, this “case-case” prediction reached a similar accuracy (AUC of 0.94) as when using the healthy blood donors as a control group (AUC of 0.91). Many of the top genes in the “case-case” classification overlap with the genes from the “case-control” classifications (Supplementary Table S3). Unique top genes in lupus nephritis defined by the “case-case” prediction are *SLC2A13*, *ZMIZ1*, *TRIB1*, *RASGRP3*, *RMI2*, and *IPMK*. *RASGRP3* has so far been associated with SLE only in Asian populations^27^, where the incidence of lupus nephritis is higher than in Caucasians. The five other genes are associated with multiple autoimmune diseases, but not with SLE.

### Random forest prediction compared to SNP association analysis

**Figure 2:**
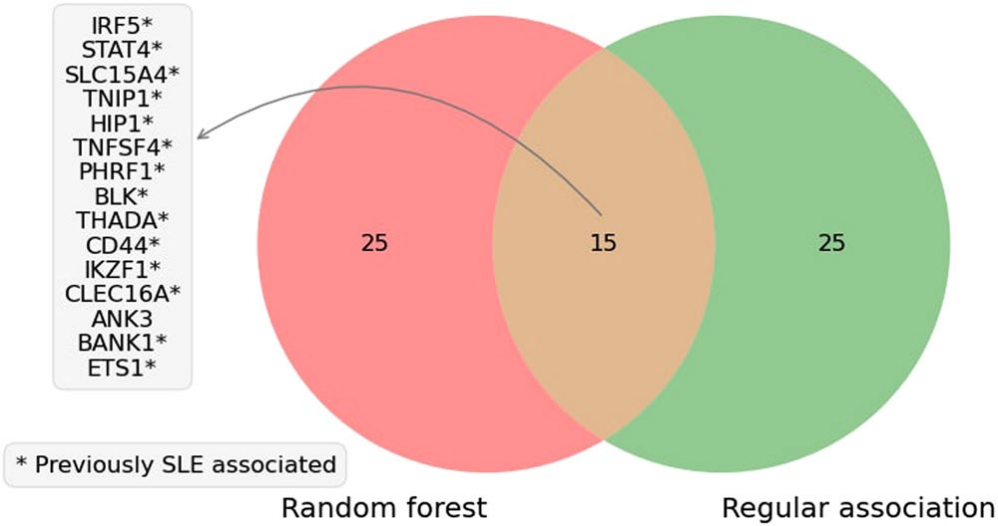
Overlapping genes. Genes overlapping between the top 40 genes defined by the random forest prediction and the regular single SNP association analysis.

The random forest method is a non-linear prediction method. It is therefore relevant to compare the performance of the random forest prediction to a logistic model, such as regular single SNP association analysis, using the same Immunochip data. The top 40 genes from the SNP association analysis (p-value < 1.29E-4), included 24 known SLE genes, 11 known autoimmune genes and 5 genes without any known connection with autoimmunity (Table 2). The genes with a nearby associated SNP with a p-value < 0.05 are reported in Supplementary Table S4. Ten of the top 40 associated genes reached statistical significance using a Bonferroni-corrected p-value < 0.05. Fifteen of the 24 SLE-associated genes among the top 40 genes over-lap with the top 40 genes predicted by the random forest classifier (Fig. 2). Notably, the 24 known SLE-associated genes obtain a relatively high rank in the random forest prediction (Table 1), with a median ranking of 19, which strengthens the validity of the random forest approach.

**Table 2:** Top 40 risk genes for SLE identified by the regular single SNP association using Immunochip genotype data from SLE patients and controls.

Predicted genes^1^	Association p-value	Association with autoimmune diseases in the GWAS catalog^4^	Differential expression in B and T cells^6,7^	Rank in random forest prediction^8^
*IRF5*	4.08E-24***	SLE, UC, IBD, RA, pSS	B > T***	15
*STAT4*	1.76E-20***	SLE, UC, IBD, RA, CD, pSS, Celiac, PBC	T > B***	3
*GTF2I*	1.05E-14***	SLE^5^, pSS	NA	3473
*NMNAT2*	5.69E-11***	SLE	Low	265
*SKAP2*	6.82E-9***	IBD, CD, T1D	B > T**	185
*ITGAM*	1.79E-8**	SLE	B > T	78
*TYK2*	3.24E-8**	SLE, UC, IBD, RA, CD, T1D, Psoriasis	B > T	106
*CFDP1*	5.41E-8**	T1D	T > B	66
*RUNX3*	7.77E-8*	CD, Celiac, AS	T > B	85
*SLC15A4*	1.09E-7*	SLE	B > T	18
*TNIP1* ^3^	3.59E-7*	SLE, IBD, Psoriasis, pSS	B > T	9
*HIP1*	4.99E-7	SLE	B > T	20
*TNFSF4*	5.28E-7	SLE, CD, RA, Celiac, MS	Low	17
*PHRF1* ^3^	6.67E-7	SLE	T > B	27
*BLK*	9.35E-7	SLE, RA, KD, pSS	B > T***	1
*PLEKHH2 THADA* ^2^	1.15E-6	SLE^5^, CD, IBD, MS	Low	10
*CD44*	1.67E-6	SLE, Vitiligo	T > B	14
*IKZF1* ^3^	2.18E-6	SLE, IBD, CD, UC	NA	12
*CLEC16A*	3.53E-6	SLE, IBD, UC, T1D, MS, CD, Psoriasis	B > T	2
*MIEN1 IKZF3* ^2^	4.19E-6	SLE, UC, CD, IBD, PBC	B > T	566
*IL10* ^3^	6.71E-6	SLE, IBD, UC, T1B, CD	T > B	122
*ENOX1 LACC1* ^2,3^	7.19E-6	IBD, CD	Low	263
*B4GALT6*	1.86E-5	New	Low	458
*ANK3* ^3^	1.89E-5	New	T > B	6
*CRB1*	1.99E-5	SLE, IBD, UC, CD	Low	119
*IFIH1*	2.93E-5	SLE, IBD, UC, T1D, Vitiligo, Psoriasis	B > T	908
*SERBP1*	3.19E-5	CD	T > B	83
*PTPN11*	3.38E-5	RA, T1D	T > B	102
*BANK1*	3.90E-5	SLE, IBD, CD	B > T***	7
*MCM6*	4.70E-5	New	T > B*	767
*RASGRP1* ^3^	6.00E-5	IBD, CD, T1D, RA	T > B**	149
*UBE2L3*	6.02E-5	SLE, IBD, CD, RA, Celiac	T > B	345
*ETS1*	6.77E-5	SLE, RA, Celiac, Psoriasis	T > B	4
*CCDC189 PRSS53* ^2^ *FBXL19* ^2^	8.63E-5	Psoriasis	Low	2054
*SLU7 PTTG1* ^2^	9.36E-5	SLE	B > T	195
*LILRB3*	9.81E-5	New	Low	392
*PHTF1*	9.85E-5	CD, T1D, RA, Vitiligo	B > T	306
*NAA25*	9.97E-5	T1D	B > T	1098
*LCT*	1.16E-4	New	Low	1475
*GSDMA*	1.29E-4	IBD, UC, CD, T1D, RA	T > B	228

^1^Human leukocyte antigen (HLA) genes not included, ^2^Alternative candidate autoimmunity gene in the region reported in the GWAS catalog or functional studies, ^3^Cis-regulatory SNP with significant association with allele-specific gene expression in B or T cells, ^4^SLE = systemic lupus erythematosus, RA = rheumatoid arthritis, IBD = inflammatory bowel disease, CD = Crohn’s disease, T1D = diabetes mellitus type 1, MS = multiple sclerosis, PBC = primary biliary cirrhosis, UC = ulcerative colitis, KD = Kawasaki disease, pSS = primary Sjögren’s syndrome, New = previously unknown SLE risk gene, ^5^Langefeld, C. D. *et al*. Transancestral mapping and genetic load in systemic lupus erythematosus, submitted manuscript, ^6^Genes are annotated according to their expression level in B or T cells based on RNA-sequencing data, ^7^Low = Expression below 1 FPKM for both cell types, ^8^Gene ranking in the random forest prediction, ^*^Bonferroni corrected p-value < 0.05, ^**^Bonferroni corrected p-value < 0.01, ^***^Bonferroni corrected p-value < 0.001.

### Functional validation in B and T cells

**Figure 3:**
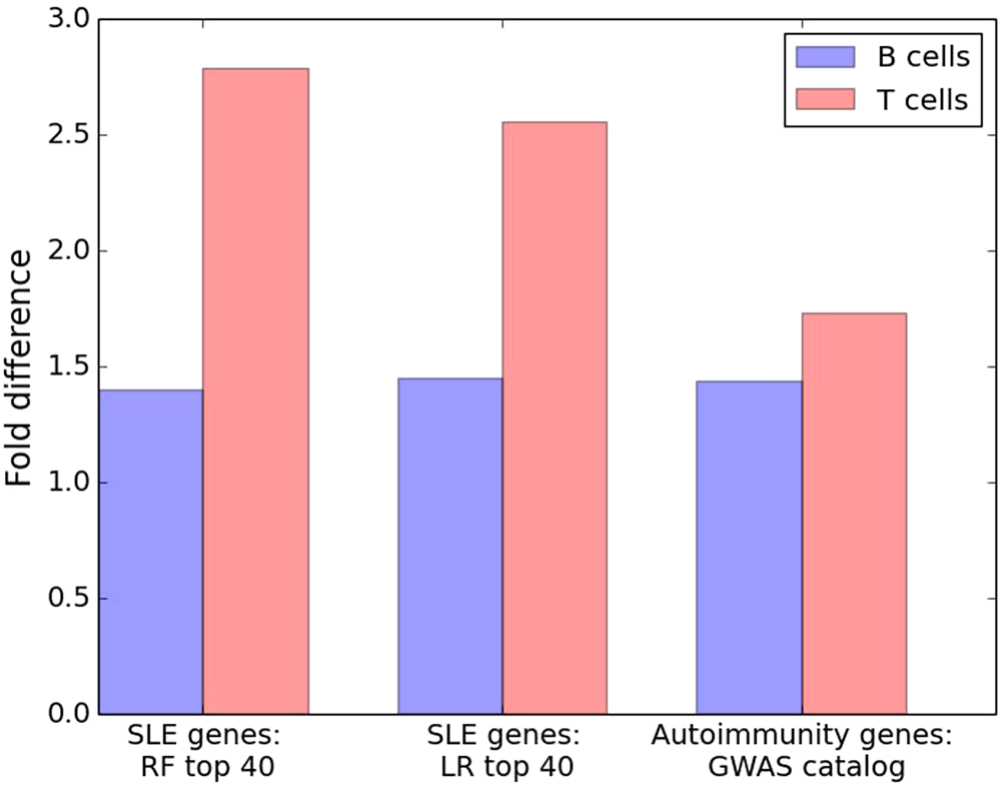
Over-representation of genes with allele-specific expression (ASE) in disease associated genes. Fold difference of expressed predicted SLE genes and autoimmunity associated genes with ASE in more than 80% of the individuals compared to all other genes. The risk genes in T-cells were significantly overrepresented in all gene sets, with the top 40 genes from random forest classification (p = 0.0079), top 40 genes from logistic regression (p = 0.015), and autoimmunity associated genes (p < 0.0001). Additionally, the enrichment of the autoimmunity associated risk genes in B-cells was also significant (p = 0.007).

We used expression patterns of the genes high-lighted by random forest classification as a functional validation in B cells and T cells from healthy donors. Allele-specific expression of a gene in a relevant cell type or tissue gives information on *cis*-acting regulation of gene expression and may serve as a guide to genes that are involved in a disease^28^. For this purpose we determined ASE of 3,000 genes in B cell and T cell samples from ~50 blood donors using genotype data from the Immunochip. We identified 739 genes in B cells and 752 genes in T cells with detectable ASE (Supplementary Table S5). Genes associated with autoimmune diseases and the top 40 genes predicted as risk genes for SLE by the random forest classifier, were over-represented in both B cells and T cells when comparing genes with ASE in at least 80% of the individual compared to all other genes. However, in the SLE top genes the over-representations was only significant in T-cells (Fig. 3). The over-representation of ASE in the risk genes predicted by random forests suggests a functional role for the predicted genes in SLE due to *cis*-regulatory SNPs (*cis*-rSNPs).

Next we mapped *cis*-rSNPs using ASE calculated from the Immunochip data of the top 40 genes from the random forest prediction. We found that 30 out of the 40 top genes were expressed in B cells or T cells (Table 2), and of these the expression of six genes was regulated by *cis*-rSNPs (Bonferroni corrected p-value < 0.05) (Table 1). Six SLE associated genes appeared to be regulated by *cis*-rSNPs: *IKZF1*, *NCF2*, *IL12A*, *TNIP1*, and *PHRF1* in B cells and *ANK3* and *PHRF1* in T cells. The *cis*-rSNPs associated with *IKZF1*, *NCF2*, *TNIP1*, *IL12A*, *PHRF1, and ANK3* are all within 12 kb of the transcription start site of the respective genes. The *cis*-rSNPs for the SLE-associated genes provides evidence for a regulatory mechanism for the allelic expression at the RNA level, and supports a functional role for *cis*-rSNPs in these genes in SLE.

**Figure 4:**
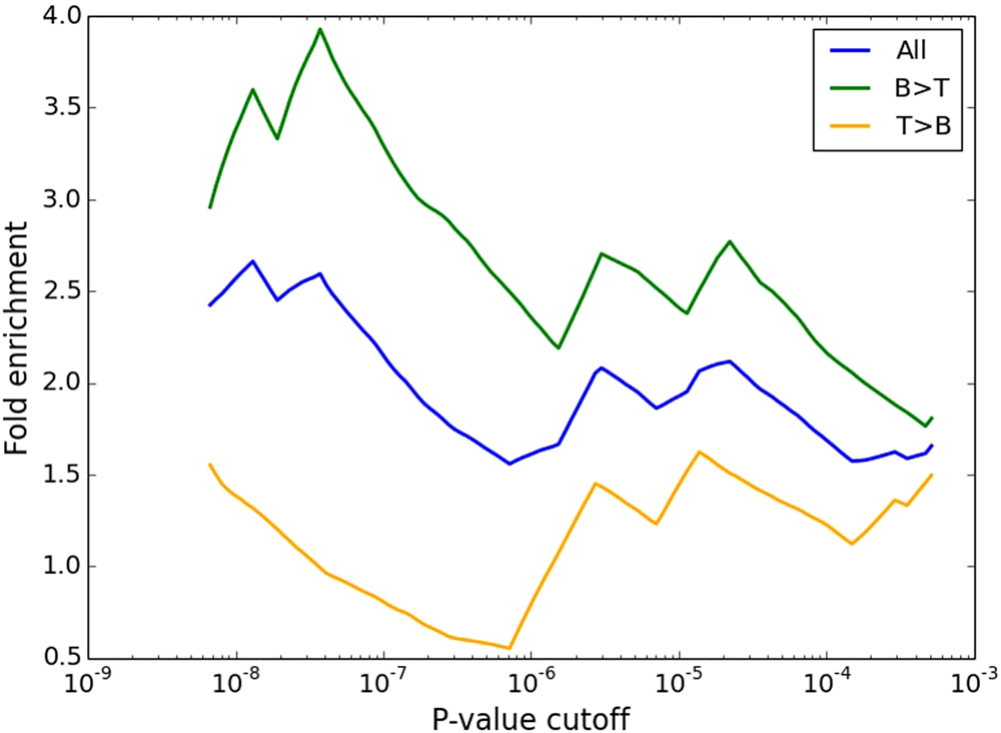
Over-representation of differentially expressed genes. Over-representation of differentially expressed genes between B cells and T cells among the top 40 genes from the random forest prediction of SLE at different significance cutoffs. Blue curve shows all genes; green curve shows genes that were expressed at a higher level in B cells than in T cells; yellow curve shows genes that were expressed at a higher level in T cells than in B cells.

To investigate expression preferences between B cells and T cells we mapped differential gene expression using RNA-sequencing data from five healthy donors. We detected differential gene expression between B cells and T cells for 1,417 genes out of 15,053 genes (RefSeq genes) after multiple testing correction (Bonferroni p < 0.05). The genes with the highest differential expression using a p-value threshold of 10^−8^ were 2-fold over-represented (p-value 0.12, Fisher’s exact test) among the top 40 genes with the highest importance scores from the random forest prediction of SLE. The over-representation was 3-fold (p-value 0.038, Fisher’s exact test) when only genes expressed at a higher level in B cells than in T cells were considered (Fig. 4). Signals of enrichment were detected for genes down to rank 500 from the random forest prediction (Supplementary Fig. S2). The over-representation of predicted risk genes for SLE genes with cell type-specific expression confirms the importance of the gene-cell type combination in the investigation of a particular disease.

Of the 30 genes that were expressed in either cell-type, 15 were expressed at a higher level in one of the cell-types. However, when only genes with significant differential expression between B cells and T cells were considered, B cell-specific genes were more common among the top 40 genes (Table 3). This pattern of preferential B cell expression was also observed for our top list of genes from the regular association analysis and for the known SLE associated genes.

**Table 3:** Difference in expression between B cells and T cells for 30 of the top 40 expressed genes from the random forest prediction.

Fold difference in expression^1^	B > X*T^1^	T > X*T^1^
X = 1	16	14
X = 2	8	5
X = 5	7	2
X = 10	6	2
X = 30	5	0
Significant difference	8	2

^1^X is the fold difference in expression between the two cell types.

## Discussion

In this study we combined machine learning with genetic association data and gene expression data to advance our understanding of SLE etiology. We focused on the top 40 most important genes predicted to confer risk for SLE by the random forest approach, and compared our results to single-SNP association data for SLE and other autoimmune diseases from the GWAS catalog. Compared to the regular single-SNP association analysis, the random forest method identified additional risk genes for SLE based on the same data from the Illumina Immunochip. Correlated variables are problematic in feature selection methods and calculations of the importance of variables. In the case of genetics, the correlation originates from linkage disequilibrium (LD) between the genetic variants. However, as the over-all gene importance score from the random forest prediction is a sum of many individual importance scores, and each individual importance score is based on an average over many trees and cross validations, the gene importance score should remain unaffected by LD.

The accuracy of the prediction of genetic risk for complex diseases varies greatly between diseases, depending on the heritability of the disease, on the uniformity of the disease phenotype, and the power and the number of investigated variants. The reported genetic predictability of SLE is low, compared to rheumatoid arthritis and several other diseases of the immune system^29^. The high level of accuracy to discriminate between SLE patients with and without lupus nephritis could be useful to identify patients at high risk of lupus nephritis before the manifestation is apparent. Risk patients could thereby be monitored more closely and possibly receive treatment at an earlier stage. At a sensitivity of 70% the specificity is 95%, which implies that with the prevalence of 24% for lupus nephritis defined in this study, a genetic test would identify 70% of the lupus nephritis patients, while 17% of the patients without lupus nephritis would be false positives. As lupus nephritis is acquired over time it is uncertain if these patients would develop or already have developed lupus nephritis after the time of phenotype data collection or if they are true false positives. Approximately 1,000 SNPs were selected in each fold of the random forest classification, making this a technically feasible test to use in a clinical setting. The relatively high prediction success in lupus nephritis probably originated from the comparative homogeneity of this subgroup of SLE patients compared to the entire group of SLE patients.

Although the number of samples is relatively high in our study, it is too small for detecting significant association signals to common SNPs with low risk and to rare SNPs with moderate risk. Also, the Immunochip is not a classical GWAS SNP-chip as it only targets autoimmunity loci. Thus the classifier could miss relevant genes for SLE that are not included on the chip. Novel genes that we identified should be subjected to independent replication as confirmation. Including other immune cell-types than B and T cells would allow more comprehensive detection of functional risk-SNPs for SLE.

Our data confirms a strong involvement of B cells in the pathogenesis of SLE. We observed an enrichment of genes among the top 40 predicted risk genes for SLE that were expressed at significantly higher level in B cells than in T cells, compared to all genes on the Immunochip (p-value 0.024, Fisher’s exact test). For example, the *ZNF804A* gene was expressed at a several-fold higher level in B cells than in T cells, which combined with functional evidence from the literature^21–24^ renders *ZNF804A* a strong novel candidate gene for SLE. For lower ranked genes the relative expression levels in B cells and T cells were equally distributed. In our study we observed an over-representation of SLE and autoimmunity genes for each of the three measures related to regulation of gene expression. One fourth of the top 40 predicted risk genes for SLE were differentially expressed in B cells versus T cells, for 15% of the risk genes we detected an associated *cis*-rSNP in at least one of the cell types, 30% of the genes had measurable ASE. In total 53% of the risk genes for SLE displayed one of these gene regulatory features, which is an enrichment compared to the expected frequency of 33% for randomly chosen genes (p-value 0.0109, Fisher’s exact test). This observation confirms from a new perspective that genes with cell type-specific regulation are more prone to be involved in SLE and other autoimmune diseases, where the risk of a gene being involved in disease is not only dependent on its function, but also on its regulatory control.

## Methods

### DNA Samples

DNA was extracted from peripheral whole blood of 1,411 SLE patients visiting the rheumatology clinics in Uppsala, Karolinska (Stockholm), Lund, Linköping and the four northern-most counties in Sweden. All patients were examined by a rheumatologist and medical records were reviewed. Control DNA was extracted from whole blood of 3,361 healthy volunteer blood donors visiting the university hospital in Uppsala (Uppsala Bioresource), Lund and Stockholm (Karolinska). SLE patients and blood donors provided informed consent to participate in the study, and the study was approved by the Regional Ethics Committees of the involved institutions. The study did not include any *in vivo* experiments on humans. The patients (included in the study) were 87% female, of Caucasian origin, and on average 36 years old at SLE onset. The patients fulfilled at least four American College of Rheumatology (ACR) 1982 criteria for SLE^18^, with the exception of eight patients who fulfilled the Fries criteria for SLE^30^. A total of 274 patients fulfilled the ACR-82 criterion for lupus nephritis. Healthy blood donors were 70% female and had an average age of 43 years at the time of blood donation.

### Isolation of human B and T cells

CD19+ B cells from 53 samples and CD3+ T cells from 54 samples were fractionated from buffy coats of 60 healthy voluntary blood donors from Uppsala by Ficoll-Hypaque (GE Healthcare) density-gradient centrifugation for isolation of PBMCs, followed by positive selection with a cell type-specific antibody (Miltenyi Biotec). Purity of the isolated cell population (>95%) was confirmed by control sampling by flow cytometry (FACSCanto II, BD Biosciences).

### Genotyping

DNA samples from SLE patients and controls were genotyped using the Illumina Infinium assay on the Immunochip, which detects about 200,000 SNPs selected based on GWAS of diseases of the immune system^13, 14^. Genotyping was performed by the SNP&SEQ Technology Platform at Uppsala University, Sweden.

The genotype data was first subjected to quality control (QC) on the sample level, whereby samples from second-degree or closer relatives, samples that did not cluster with Europeans in principal component analysis (PCA), samples with heterozygosity rates exceeding five standard deviation from the average and samples with genotype call-rates below 95% were removed. After these QC parameters, 1,160 SLE patients and 2,711 controls remained for further analysis. Genotype data from the sex-chromosomes and from insertion-deletions were excluded. The QC on the SNP level removed SNPs with an average sample call-rate below 98%, SNPs with a Hardy-Weinberg equilibrium (HWE) p-value below 10E-4, and SNPs with a minor allele frequency below 1%. After QC, genotype data for 134,523 SNPs remained for further analysis. Genes in the HLA region are not reported in the association analysis and the random forest prediction due to high gene density and strong LD making it very hard to determine the causative gene.

### SNP association analysis

The genotype data for individual SNPs from the Immunochip was analyzed for association with SLE using logistic regression in PLINK version 1.07^31^. SNPs were annotated to overlapping genes or to the closest downstream gene within 100 kb for intergenic SNPs. When SNPs in high linkage disequilibrium (LD) were associated with several genes, only the SNP-gene combination with the lowest association p-value was kept for further analysis. For associated SNPs that showed a significant difference in number of missing genotypes between controls and patients (p-value < 0.05) or a HWE p-value < 0.05, the genotype cluster plots were inspected manually and SNPs were filtered out if the called genotypes appeared to be based on low quality data.

The accuracy of the logistic regression to predict disease status was assessed by splitting the association data into training data sets (80%) and test data sets (20%) in five different folds. Only SNPs with an association p-value < 0.01 were used in the prediction. Based on the training data, a risk score was calculated for the test data defined by the difference between the total number of risk and protective alleles in each individual. Using the risk score, the predictive performance was evaluated in the merged test data from the five folds using the AUC measure.

### Random forest predictions

Prediction of SLE status and calculation of a gene importance score based on the genotype data from the Immunochip was performed using a random forest machine learning method^8^. The computations were run using the R package Emil (Evaluation of Modeling without Information Leakage, Christofer L Bäcklin, Mats G Gustafsson (2014), version 1.1-6.), which in turn uses the RandomForest R-package^32^.

SLE status was predicted based on genotype data in three iterations with five cross-validation folds per iteration, where each of the 15 cross-validation runs used 80% of the data for training of the classifier and 20% for testing, using on average the 3,000 most informative SNPs per classification fold. The SNPs were selected based on training data only within each fold. Fisher’s exact test was calculated for each site and only sites with a p-value < 0.01 were included.

The number of variables selected per tree (mtry) and number of trees (tree) for the random forest algorithm were set to 300 and 1,000, respectively. A larger number of trees did not improve the prediction. mtry was set to approximately 0.1 times the number of selected variables, which is recommended for sparse data^11^.

The Gini importance measure was used to determine the importance of each SNP. This measure is calculated by the random forest algorithm and describes the classification performance of a variable averaged over all trees and nodes where the variable was used. An importance score for each gene was defined by summing the Gini importance measure of individual SNPs within a gene and its 10 kb flanking regions. Finally, the summed importance score for each region was averaged over the 15 cross validation folds. Gene regions were obtained from the Reference sequence (RefSeq) database at NCBI^33^. We also searched for autoimmune disease annotated genes close to high scoring genes without an autoimmune disease annotation in the GWAS catalog. When relevant genes were found, the gene region was expanded into a gene region including all relevant genes as candidate genes.

### Heritability estimates

To determine the heritability explained by the different models, we used the AUC for the respective statistical model in conjunction with disease prevalence and sibling recurrence risk of SLE^29^. The SLE prevalence in the Swedish population was set to 68 in 100,000^34^ and the sibling recurrence risk for SLE that is 20 times higher than for non-siblings. For lupus nephritis the prevalence in the Swedish population was set to 23 in 100,000^34^.

### Allele-specific gene expression analysis

Fractionated B cells from peripheral blood of 53 healthy donors and T cells from 54 donors were subjected to ASE analysis by SNP genotyping as described previously for human monocytes^15^. DNA and RNA were prepared from B and T cells using the AllPrep DNA/RNA Mini Kit (Qiagen). cDNA was synthesized from 1–5 µg of RNA using the SuperScript Double-Stranded cDNA Synthesis Kit (Invitrogen). Double-stranded cDNA was purified using the MinElute PCR purification kit (Qiagen).

ASE levels can be determined by RNA-sequencing^35–37^ or as in this study by quantitative genotyping of heterozygous SNPs on the RNA level^38^. Genomic DNA (gDNA) and complementary DNA (cDNA) from B cells and T cells were genotyped in parallel using the Immunochip. Genotypes were called in gDNA using the Genome Studio version 2009.2 (Illumina) with a call rate of 99% as the threshold for SNP genotype calls and 98% sample success rate. SNPs were further filtered on deviations from HWE with a p-value cutoff of 10^−6^ (Chi-squared test). ASE-levels were determined using the genotype data calculated for each gene region as described in Almlöf *et al*.^15^. In short, the ASE-levels were calculated for each heterozygous SNP as the difference in normalized allele fractions between cDNA and gDNA: [Allele1cDNA/(Allele1cDNA + Allele2cDNA)] − [Allele1gDNA/(Allele1gDNA + Allele2gDNA)]. *Cis* regulatory SNPs (*cis-*rSNPs) were called in each gene region and 100 kb flanking regions, having at least five heterozygous SNPs with a fluorescence intensity over 5,000, by logistic regression analysis against the ASE-levels essentially as described by Ge *et al*.^39^. The analysis was performed on 2,604 gene regions in B cells and 2,582 gene regions in T cells. Additionally, an ASE-value for entire gene regions was determined using the median of the absolute ASE-levels for SNPs within each gene region for all individuals. To minimize the number of false positive signals ASE was only called in gene regions with more than 20 observations of SNP-individual combinations in expressed gene regions (Supplementary Fig. S3). A region was considered as expressed if the fluorescence intensity corresponding to one of the alleles was higher than 5,000 fluorescence units (Supplementary Fig. S4). The lower limit of the difference in allele fraction between RNA and DNA for calling ASE was set to 0.075. ASE-levels above this cut-off did not increase the signal to noise ratio and signal intensities above 5,000 only slightly increased the signal to noise ratio (Supplementary Fig. S5). In the B cell data 2,958 gene regions and in the T cell data 3,010 gene regions passed all QC criteria for calling ASE.

### RNA-sequencing

The transcriptomes of B and T cells from 5 healthy blood donors were subjected to RNA-sequencing. Ribosomal RNA (rRNA) was depleted from 1 µg of total RNA using the Ribo-Zero Magnetic Gold Kit (Epicentre). Strand-specific RNA-sequencing libraries were constructed from rRNA-depleted RNA with the ScriptSeq V2 Kit (Epicentre). The libraries were sequenced using an Illumina Hiseq. 2000 instrument using paired-end 50 bp reads, which yielded 14M-89M read pairs per sample (median 58 M). The reads were aligned with TopHat and transcript assembly was performed by Cufflinks^40^. Differential expression between B and T cells was detected using the limma R package^41, 42^ and voom normalization^43^.

### Data Availability

Genotyping summary data are available from the corresponding author on reasonable request. ASE summary data are available from the corresponding author on reasonable request. Raw and normalized FPKM values generated from RNA-sequencing data are available in the GEO repository with IDs: GSM1978773–GSM1978782 at http://www.ncbi.nlm.nih.gov/gds.

## Electronic supplementary material

Supplementary Figures S1-S5 and Tables S2,S3,S5

Supplementary Table S1

Supplementary Table S4
