# Supplementary material for: Novel risk genes for systemic lupus erythematosus predicted by random forest classification
Source: Sci Rep. 2017 Jul 24;7:6236. doi: 10.1038/s41598-017-06516-1 (PMC5524838; doi:10.1038/s41598-017-06516-1)
Supplement: Supplementary file 1 — Supplementary Figures S1-S5 and Tables S2,S3,S5 [file 41598_2017_6516_MOESM1_ESM.pdf]

Novel risk genes for systemic lupus erythematosus predicted by random forest classification

Jonas Carlsson Almlöf, Andrei Alexsson, Juliana Imgenberg-Kreuz, Lina Sylwan, Christofer Bäcklin, Dag Leonard, Gunnel Nordmark, Karolina Tandre, Maija-Leena Eloranta, Leonid Padyukov, Christine Bengtsson, Andreas Jönsen, Solbritt Rantapää Dahlqvist, Christopher Sjöwall, Anders A. Bengtsson, Iva Gunnarsson, Elisabet Svenungsson, Lars Rönnblom' Johanna K. Sandling, and Ann-Christine Syvänen

Supplementary Fig. S1 Distribution of Gini importance scores for the genes in the Random forest prediction. The y-axis shows the score using a logarithmic scale and the x-axis the gene ranking.

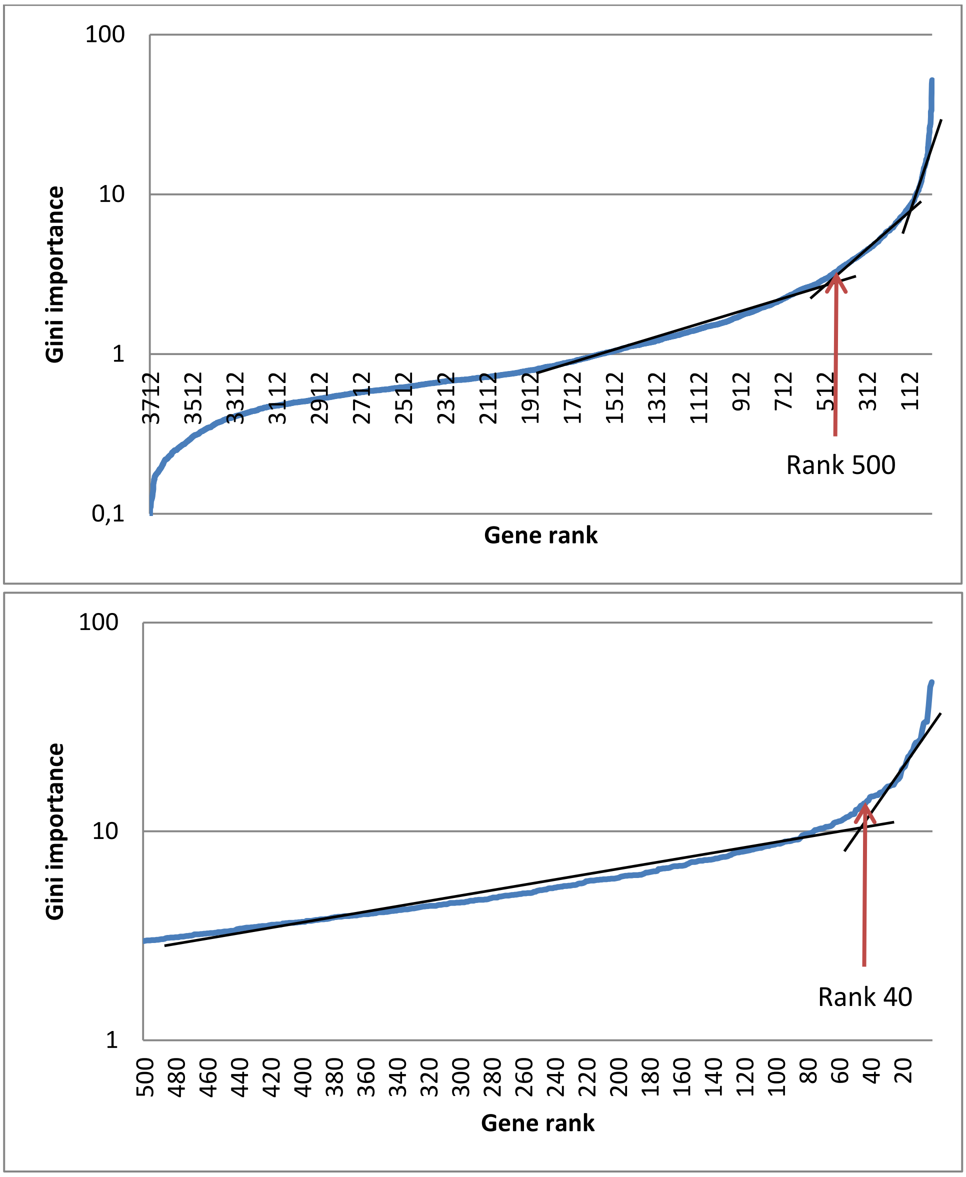

Supplementary Fig. S2 Overrepresentation of differentially expressed genes between B cells and T cells from the SLE random forest prediction using different significance cutoffs for differential expression. Blue curve shows all genes; green curve shows genes that were expressed at a higher level in B cells than in T cells; yellow curve shows genes that were expressed at a higher level in T cells than in B cells. A) Top 41-100 genes compared to all genes. B) Top 101-500 genes compared to all genes. C) Top 501-1,000 genes compared to all genes.

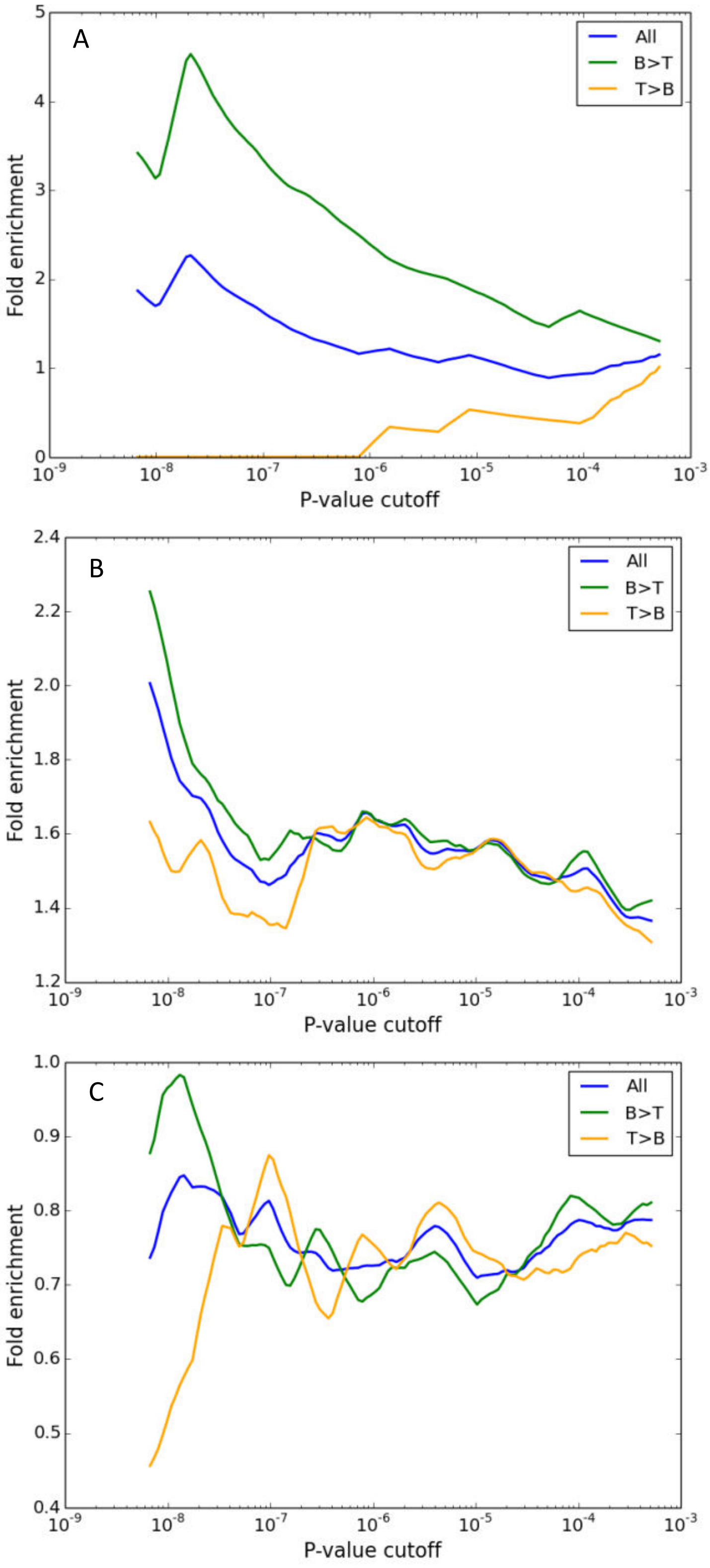

Supplementary Fig. S3 Average variance in allele-specific expression level depending on the number of observations of SNP-individual combinations. The cut-off used in the analysis was set to more than 20 observations (see M&M).

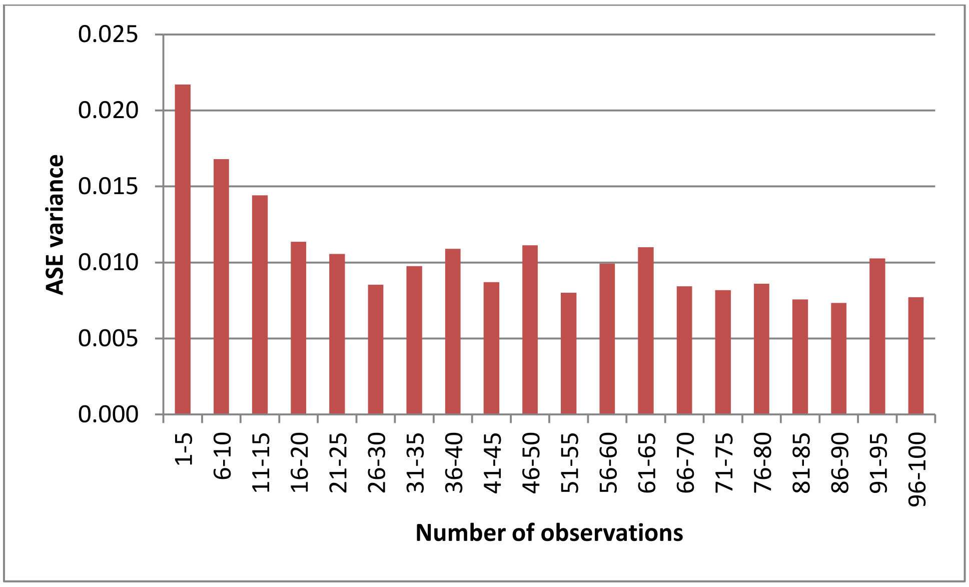

Supplementary Fig. S4 Percentage of allele-specific expression (ASE) observations of SNP individual combinations where the background signal in a homozygous individual was in the same or higher intensity bin than the expressed allele of a heterozygous individual at the same genomic position. 5,000 fluorescence units was used as cut-off for calling ASE (see M&M). Data combined from B cells and T cells.

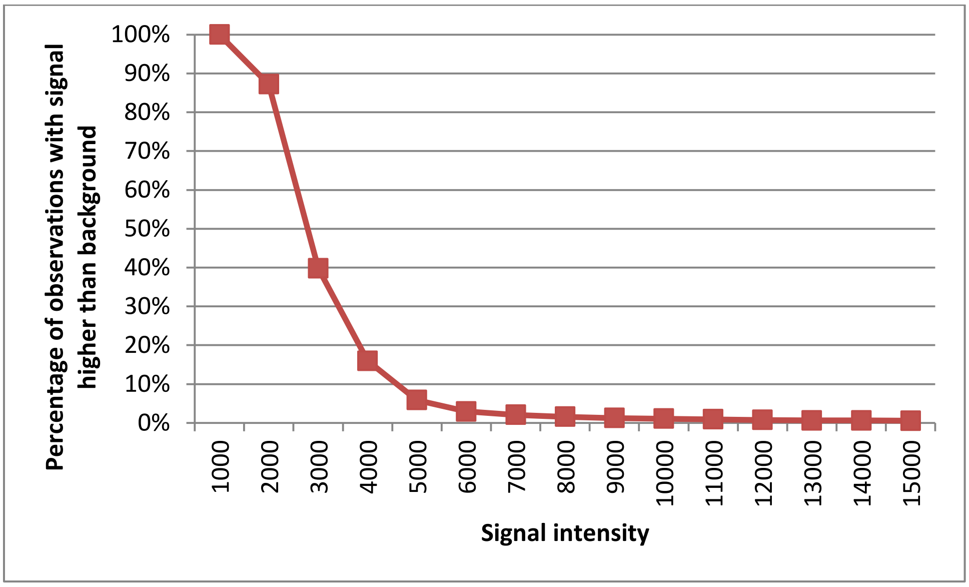

Supplementary Fig. S5 Percentage of SNPs with average allele-specific expression (ASE) levels that were higher in heterozygous samples than in homozygous samples at different ASE-level cutoffs. An ASE-level of 0.075 was used as a cut-off for calling ASE (see M&M). Data combined from B cells and T cells.

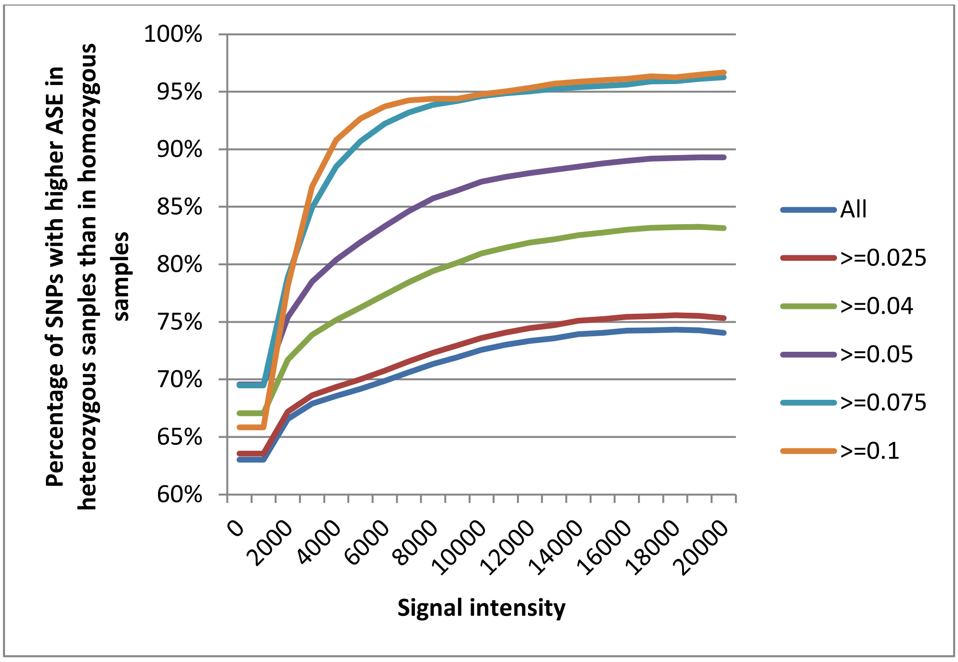

Supplementary Table S2 Genes in the top 40 random forest prediction with an autoimmune association other than SLE and its possible function in the SLE pathogenesis.

Gene	SLE related function found in literature	Reference
PSMG1	NA	
PTGER4	PGE2 is a potent IFN-α inhibitor in PDCs. The effects are mainly mediated by the PG receptors EP2 and EP4 (PTGER4).	[2]
CPEB4	NA	
EGR2	Together with EGR3 is a key negative regulator of T cell activation in mice	[4]
RFX3	The expression and activity of the transcription factor RFX1 are decreased in SLE CD4+ T cells. *RFX1* affects transcription of the class II MHC genes. *RFX1* is closely related to *RFX3*.	[5]
IL1R1	Receptor for IL1A, IL1B and IL1RN. After binding to interleukin-1 associates with the co-receptor IL1RAP to form the high affinity interleukin-1 receptor complex which mediates interleukin-1-dependent activation of NF-kappa-B, MAPK and other pathways	[7]
LRRK2	Positively regulates autophagy through a calcium-dependent activation of the CaMKK/AMPK signaling pathway.	[8]
GPR183	Overexpression of GPR183 profoundly potentiated antibody-stimulated ex vivo proliferation of murine B cells compared with WT cells, whereas this was equivalently reduced for GPR183-deficient B cells. Signals constitutively via MAPK1.<br>MAPK1 is upregulated in SLE.	[9, 10]
ZMIZ1	Encodes for the zinc finger protein MIZ type 1, which is a member of the protein inhibitors of activated STAT (PIAS).<br>PIAS proteins are co-regulators of the JAK-STAT pathway<br>Can also regulate the TGF-β/SMAD signaling18 that has been clearly demonstrated to have an inhibitory effect on the immune system	[13-15]
ELMO1	ELMO is an upstream regulator of Rac1 which is a part of the NCF2/NCF4/VAV1/RAC1 NADPH oxidase complex where destabilizing mutations in NCF2 are associated to SLE.	[16, 17]
TNFSF11	Activates the antiapoptotic AKT1 through a signaling complex involving c-Src and TRAF6. AKT1 is upregulated in PBMCs from SLE patients.	[10, 18]
SATB2	NA	

References

1. Drieschner N, Kerschling S, Soller JT, Rippe V, Belge G, Bullerdiek J, Nimzyk R: A domain of the thyroid adenoma associated gene (THADA) conserved in vertebrates becomes destroyed by chromosomal rearrangements observed in thyroid adenomas. Gene 2007, 403;1-2:110-7.
2. Fabricius D, Neubauer M, Mandel B, Schutz C, Viardot A, Vollmer A, Jahrsdorfer B, Debatin KM: Prostaglandin E2 inhibits IFN-alpha secretion and Th1 costimulation by human plasmacytoid dendritic cells via E-prostanoid 2 and E-prostanoid 4 receptor engagement. Journal of immunology 2010, 184;2:677-84.
3. Wolf SF, Temple PA, Kobayashi M, Young D, Dicig M, Lowe L, Dzialo R, Fitz L, Ferenz C, Hewick RM et al: Cloning of cDNA for natural killer cell stimulatory factor, a heterodimeric cytokine with multiple biologic effects on T and natural killer cells. Journal of immunology 1991, 146;9:3074-81.
4. Safford M, Collins S, Lutz MA, Allen A, Huang CT, Kowalski J, Blackford A, Horton MR, Drake C, Schwartz RH et al: Egr-2 and Egr-3 are negative regulators of T cell activation. Nature immunology 2005, 6;5:472-80.
5. Zhao M, Sun Y, Gao F, Wu X, Tang J, Yin H, Luo Y, Richardson B, Lu Q: Epigenetics and SLE: RFX1 downregulation causes CD11a and CD70 overexpression by altering epigenetic modifications in lupus CD4+ T cells. Journal of autoimmunity 2010, 35;1:58-69.
6. Hayden MS, West AP, Ghosh S: NF-kappaB and the immune response. Oncogene 2006, 25;51:6758-80.
7. Slack JL, Schooley K, Bonnert TP, Mitcham JL, Qwarnstrom EE, Sims JE, Dower SK: Identification of two major sites in the type I interleukin-1 receptor cytoplasmic region responsible for coupling to pro-inflammatory signaling pathways. The Journal of biological chemistry 2000, 275;7:4670-8.
8. Gomez-Suaga P, Luzon-Toro B, Churamani D, Zhang L, Bloor-Young D, Patel S, Woodman PG, Churchill GC, Hilfiker S: Leucine-rich repeat kinase 2 regulates autophagy through a calcium-dependent pathway involving NAADP. Hum Mol Genet 2012, 21;3:511-25.
9. Benned-Jensen T, Smethurst C, Holst PJ, Page KR, Sauls H, Sivertsen B, Schwartz TW, Blanchard A, Jepras R, Rosenkilde MM: Ligand modulation of the Epstein-Barr virus-induced seven-transmembrane receptor EBI2: identification of a potent and efficacious inverse agonist. The Journal of biological chemistry 2011, 286;33:29292-302.
10. Garcia-Rodriguez S, Callejas-Rubio JL, Ortego-Centeno N, Zumaquero E, Rios-Fernandez R, Arias-Santiago S, Navarro P, Sancho J, Zubiaur M: Altered AKT1 and MAPK1 gene expression on peripheral blood mononuclear cells and correlation with T-helper-transcription factors in systemic lupus erythematosus patients. Mediators of inflammation 2012, 2012:495934.
11. Colombo T, Farina L, Macino G, Paci P: PVT1: a rising star among oncogenic long noncoding RNAs. BioMed research international 2015, 2015:304208.
12. Sarhan RA, Aboelenein HR, Sourour SK, Fawzy IO, Salah S, Abdelaziz AI: Targeting E2F1 and c-Myc expression by microRNA-17-5p represses interferon-stimulated gene MxA in peripheral blood mononuclear cells of pediatric systemic lupus erythematosus patients. Discovery medicine 2015, 19;107:419-25.
13. Shuai K: Modulation of STAT signaling by STAT-interacting proteins. Oncogene 2000, 19;21:2638-44.
14. Li X, Thyssen G, Beliakoff J, Sun Z: The novel PIAS-like protein hZimp10 enhances Smad transcriptional activity. The Journal of biological chemistry 2006, 281;33:23748-56.
15. Wan YY, Flavell RA: TGF-beta and regulatory T cell in immunity and autoimmunity. Journal of clinical immunology 2008, 28;6:647-59.
16. Gumienny TL, Brugnera E, Tosello-Trampont AC, Kinchen JM, Haney LB, Nishiwaki K, Walk SF, Nemergut ME, Macara IG, Francis R et al: CED-12/ELMO, a novel member of the CrkII/Dock180/Rac pathway, is required for phagocytosis and cell migration. Cell 2001, 107;1:27-41.

17. Armstrong DL, Eisenstein M, Zidovetzki R, Jacob CO: Systemic lupus erythematosus-associated neutrophil cytosolic factor 2 mutation affects the structure of NADPH oxidase complex. The Journal of biological chemistry 2015, 290;20:12595-602.
18. Wong BR, Besser D, Kim N, Arron JR, Vologodskaia M, Hanafusa H, Choi Y: TRANCE, a TNF family member, activates Akt/PKB through a signaling complex involving TRAF6 and c-Src. Mol Cell 1999, 4;6:1041-9.

Supplementary Table S3 Top 40 risk genes for lupus nephritis using a “case-case” random forest classifier on Immunochip genotype data from SLE patients with and without lupus nephritis.

**Predicted gene**	**Rank in random forest prediction**
CLEC16A	26
THADA	5
XKR6	25
ZNF804A	1
IL2RA	44
GLIS3	19
SLC2A13	432
ANK3	37
SATB1-AS1	45
BLK	10
BANK1	3
ZMIZ1	171
TRIB1	110
PRKCQ	16
PTGER4	72
PVT1	39
RASGRP3	279
NOS2	38
UBE2E3	74
RMI2	583
IPMK	272
FAM167A	27
PTPN2	124
ZNF365	28
ETS1	14
CPEB4	79
VENTXP7	820
SIGLEC6	>3712
RBFOX1	352
MAPKAPK2	527
KIF21B	2256
USP12	59
GLB1	75
TNIP1	6
PAPOLG	741
TGFBR3	769
LYN	69
TH	13
RASGRP1	2584
SMAD3	542

Supplementary Table S5 Summary statistics for allele-specific gene expression in B cells and T cells.

	**Number of tested genes**	**Percentage of genes with ASE > 0.075**
**B cells**		
All tested genes	2958	25%
All genes in GWAS catalog	1467	26%
All genes in GWAS catalog associated with autoimmunity	417	30%
SLE associated genes in GWAS catalog	34	18%
Top 40 random forest predicted genes	35	20%
**T cells**		
All tested genes	3010	26%
All genes in GWAS catalog	1483	27%
All autoimmune associated genes in GWAS catalog	481	27%
SLE associated genes in GWAS catalog	35	31%
Top 40 random predicted genes	35	29%
